# Treatment of the Dental Pulp

**Published:** 1881-12

**Authors:** A. G. Bouton

**Affiliations:** Savannah, Ga.


### ARTICLE IV

### *Treatment of the Dental Pulp.*

BY A. G. BOUTON, D. D. S., SAVANNAH, GA.

[Read before the Georgia State Dental Society, May 12, 1881.]

The desirability of retaining the vitality of the pulp is too manifestly simple a question to require argument. The results of the common method of devitalizing and removing the pulp, even when properly and very carefully performed, are not frequently of an entirely satisfactory character. While comparative comfort may be secured to a tooth after the pulp chamber has been so completely filled as to prevent this cavity from becoming the receptacle of effusions, thereby excluding the irritating effects of the expansion of gasses occasioned by the decomposition of these effusions, it cannot be denied that the tooth has a sense of weakness; that discoloration frequently ensues; that it is liable to attacks of inflammation of the peridental membrane; that it is more liable to caries; that the effects of carious action are more disastrous; and finally, that even when unattacked by caries, from the gradual weakness of cohesion, premature loss is liable to happen by fracture. These reasons alone should be sufficient to constrain us to the employment of some conservative treatment which would promise the preservation of a moderate presentage of pulps.

When a tooth has become subject to carious action, it is, as soon as the decomposition process reaches the dentine, made manifest that the pulp is cognizant of some derangement, and is more or less irritated, if not, certainly its dependencies, the contents of the tubuli, and commence the process of protecting the pulp from serious irritation by calcification of their terminal filaments. This beautiful and regular action may reasonably be accepted as one of the most conclusive facts of the power of the pulp to protect itself ; of its power of self-preservation and repair. No sooner is this zone of defense invaded than a new one is completed, and so on continually until the pulp is so nearly approached as to bring on a new train of symptoms and new conditions.

The derangement of the dental pulp which follows the unchecked course of caries, may properly be divided into two classes.

1st. Conditions induced by the actual contact of caries, the pulp remaining covered by the gelatinous residue.

2d. Conditions caused by the complete exposure to external irritants.

1st condition.—This condition is often exceedingly difficult to diagnose; frequently it will be found that in cases when full exposure was not expected after a more careful examination at one of the corners, slight uncovering has been effected. One of the chief causes of this difficulty springs from the fact that the pulp may be reached by the carious action, and remain in contact with the decay for a considerable period without its health being disturbed. In such cases we should operate with great care. If, after opening the cavity, defining the margins, and removing carefully the greater part of the caries, it is found from the extent of the cavity that the pulp may, in some degree, be implicated, and no spontaneous pain is found to have existed, and in filling the cavity for the moment with some non-conductor, there is no response to the application of cold, and if the pulp is not hurt by pressure with a blunt instrument upon a pellet of cotton, it may be safely considered that the pulp, however nearly, is not fully in contact with the caries. And just here let me say that even after satisfying myself that the pulp is not fully in contact with caries or, in other words, is not fully exposed, I do not advocate the entire removal of all decaying dentine. When caries has extended beyond the last layer of consolidated dentine a somewhat disturbed state of the pulp arises. This increased irritation is probably occasioned by some portion of the fluids of the cavity finding their way through the remaining portion of dentine. At this stage, when the pulp is so nearly reached, the moment quickly arrives when a number of the tubuli are without obstruction, and when their contents should be viewed, in the field of treatment as the pulp proper.

The unnecessary removal of the last layer of decay by its rudeness, however carefully performed, drags outward the tubuli contents and it is a very precarious step at this stage of caries. But to return. Now, on the other hand, should there have been pain on the side of the face including the tooth, although such pain may have been transitory, and if, on applying cold to the enamel of the tooth, the cavity being stopped as above referred to, there should be complaint of the cold, it may be concluded that the pulp is fully reached. If any uncertainty may exist, it will be decided by the application of pressure as above stated.

The extreme sensibility of the tooth to cold is one of the most characteristic symptoms of irritation of the pulp. This diagnostic symptom becomes of extreme use in the after treatment as a means of determining whether the irritation of the pulp is increasing or declining. It appears to be due to the terminal filaments of the tubuli contents being excessively excited by the irritation which has effected the nervous elements of the pulp. It will be found that in most cases the pulp has remained exposed probably for weeks, perhaps for months, without the occurrence of any more pain than will occasionally call the attention of the mind to the condition of the tooth. In every such case, the utmost attention from a healthy state which is observable is a slight hyperæmia one. The fact that the pulp will remain healthy for so long a time is of the greatest importance, and indicates what has agreed with my own experience, that the dental pulp when carefully and intelligently handled, there is but little danger of exciting disturbance.

As to the treatment of these simple exposures after carefully removing the softer caries and nearly uncovering the pulp, my practice for some time has been at this step, in case no pain has been previously experienced which could be referred to the tooth under treatment, to apply dilute carbolic acid, not stronger than ten per cent. to the remaining layer of half decomposed dentine. I then apply a thick paste of Lacto-Phosphate of Lime, and fill the cavity with Oxyphosphate cement. If pain has for several days existed, however slight, the nervous irritation has induced hyperæmia, and this condition must be relieved, or else the attempt to operate will but further the irritation. With a light hand, without wounding the pulp, the ultimate layer of caries should be removed and some remedy applied to relieve the hyperæmia and stop the determination of blood. For this I have had great satisfaction from the use of Aconite; it is to nervous tissue a sedative, and at the same time possesses specific action upon the capillary system. The formula I mostly use is Tincture Aconite rad,

Chloroform equal parts. This is applied to the point of exposure upon a pellet of cotton. The cavity during this, and subsequent steps, should be kept perfectly dry, by the use of the rubber dam. After removing the Aconite, I made an application of Dilute Carbolic Acid as before mentioned, and when the excess of this is taken up by bibulous paper, flow over the point of exposure and bottom of the cavity a cement composed of Phosphate Lime and Oxide Zinc, equal parts, with concentrated Lactic Acid as the liquid. This cement will set in three or four minutes sufficiently hard to fill over with gold if desired. After the cement has hardened, fill the cavity with gutta-percha. Carbolic Acid, it must not be overlooked, is itself in any state of concentration beyond ten per cent. an irritant, and while a remarkable connection of fermentative processes may, when used in greater strength than indicated, become so irritating as actually to sustain the condition for which it is employed to correct, the better strength I have found to be ten per cent., the menstrum glycerine. Should the pulp have been wounded, Tincture Calendula, one part to three of water, should be applied immediately. If congestion of the peridental membrane is found present, or takes place during the course of treatment, the gum should be slightly scarified in the neighborhood of the tooth, and Aconite painted upon the scarified surface, or carefully applied on a pellet of cotton. In cases where the long continued disturbance will not immediately be removed, I have had most success in correcting this condition, by the action of Aconite. It should be applied on a pellet of cotton and the cavity hermetically sealed until the next day.

It is important when the pulp has been completely denuded of all its covering that it be not allowed to remain unprotected from the air and the fluids of the mouth ; for this reason the edges of the cavity should be so well prepared as to ensure the retention of the temporary filling, and for the same reason in cases when the disturbance has been too great to permit an immediate closure, the cavity must be so far sealed as to exclude all outer influences.

It is essential, and cannot be too strongly insisted upon, that the use of concentrated escharotics and astringents should be avoided. That which is healthy does not need modification, and that which has but slightly departed from the normal standard of health needs but simple treatment.

It will not fail to be observed that one of the ends kept in view in the treatment here indicated for this class of cases is the preservation of the integrity of the dentinal cells to maintain their function by careful manipulation and to avoid destroying them by over treatment, by the use of strong escharotics. The question now arises, what is to be the ultimate result of this treatment. Will the pulp remain alive without histological changes, or will it in time commence and complete such a reparative effort as will permit a permanent completion of the operation.

It will be naturally expected that if the pulp remains in a healthy condition beneath the filling, that some reparative effort will take place, that at length the conversion of some of the dentinal cells into completed dentine will be effected. I have in a number of cases seen when this change has taken place, but in all cases in which I have been successful in saving the pulp alive, I cannot say with positiveness that this change has actually occurred, for unless pain has been experienced after the preparatory treatment, nothing more is needed beyond maintaining the proper fullness of the temporary filling, and when filling permanently, I seldom remove more of the cement than is necessary to properly introduce the permanent filling. Facts confirming this change, however, are so abundant that no preservation of them is necessary.

A period of from three to six months should elapse before completing the treatment by a permanent filling.

2d. condition.—When ultimate denudation of the pulp has taken place, and has continued for some time, the difficulties of the treatment must be very much enhanced; under these circumstances the surface exposed to the air is much more highly charged with blood than is normally the case, and in many instances is in a constant state of effusing lymph, at times pus—in other words the surface is an ulcer, and is to be treated as such. So precarious is the management of this class of cases, that no definite rules can be laid down which will be applicable to all cases. That which will at one time give relief, and permit closure of the cavity, at the next when the symptoms are similar, and conditions apparently the same, will be followed by a return of pain, indicating that effused fluids are pressing between the filling and pulp.

In these cases various irritations have asserted themselves, and probable repeated attacks of pain have been suffered; the consequence of severe congestions. Alteration of structure may have taken place, which will never permit the functions of the pulp to be normally carried on. The important changes which have taken place at the point of exposure, where it has been directly acted upon by various deleterious influences, are according to the investigations of Wedll, the German observer, of such a nature as to render questionable a proper cure. He says of them: "The dental cells over the exposed parts are obliterated. The connective tissue has suffered pulifferations. The blood-vessels are altered by serious structural changes. The nerve fibres also have been deranged by the deposition of fat panules, not only between them, but in their interior. In short, all the structural elements of the pulp undergo changes."

It would appear, then, that the destruction of the dentinal cells renders impossible a conversion of this portion of the pulp into true dentine. That these odontoblasts studding the whole surface have as their function the maintenance of connection between the deeper pulp tissue and the tubular contents, and also carry on the slow formation of dentine. But the experience of many are not wanting in confirmation of the formation of secondary dentine which at length bridges over and becomes connected with the sides of the pulp capsule.

In the general treatment of these cases I use Chloride Zinc, one part to about forty of water; apply this to the ulcerated surface of the pulp and arch the part over until the next day, when Aconite should be applied as described, and separated until the pulp has so far recovered as to permit a covering to be placed immediately upon it. For this covering the cement before mentioned should be used.

Fortunately completely denuded pulps do not frequently occur except for those who are so careless about their teeth that little can be done for them Of course each case must be "a law unto itself." But I hold that even in these doubtful cases a large enough percentage of pulps can be saved alive to warrant the attempt in all cases when the indications promise even a chance of success. And if it fails, the one resort still remains of canal treatment, but which we are not warranted in resorting to until everything else fails. The rule should be, that every exposed pulp should have a chance for life.

---

